# Deciphering Master Gene Regulators and Associated Networks of Human Mesenchymal Stromal Cells

**DOI:** 10.3390/biom10040557

**Published:** 2020-04-05

**Authors:** Elena Sánchez-Luis, Andrea Joaquín-García, Francisco J. Campos-Laborie, Fermín Sánchez-Guijo, Javier De las Rivas

**Affiliations:** 1 Bioinformatics and Functional Genomics Group, Cancer Research Center (CiC-IMBCC, CSIC/USAL/IBSAL), Consejo Superior de Investigaciones Científicas (CSIC) and University of Salamanca (USAL), 37007 Salamanca, Spain; elenasl@usal.es (E.S.-L.); andreajoaquingarcia@gmail.com (A.J.-G.); fjcamlab@gmail.com (F.J.C.-L.); 2 Bioinformatics and Cancer genomics, Wellcome Trust/Cancer Research UK Gurdon Institute, University of Cambridge, CB2 1QN Cambridge, UK; 3 Cell Therapy Area and Department of Hematology, Institute of Biomedical Research of Salamanca -Hospital Universitario de Salamanca (IBSAL-HUS) and Department of Medicine, University of Salamanca (USAL), 37007 Salamanca, Spain; ferminsg@usal.es

**Keywords:** mesenchymal stromal cells, transcription factor, regulons, master regulators, gene networks, transcriptomics, bioinformatic, meta-analysis

## Abstract

Mesenchymal Stromal Cells (MSC) are multipotent cells characterized by self-renewal, multilineage differentiation, and immunomodulatory properties. To obtain a gene regulatory profile of human MSCs, we generated a compendium of more than two hundred cell samples with genome-wide expression data, including a homogeneous set of 93 samples of five related primary cell types: bone marrow mesenchymal stem cells (BM-MSC), hematopoietic stem cells (HSC), lymphocytes (LYM), fibroblasts (FIB), and osteoblasts (OSTB). All these samples were integrated to generate a regulatory gene network using the algorithm ARACNe (Algorithm for the Reconstruction of Accurate Cellular Networks; based on mutual information), that finds *regulons* (groups of target genes regulated by transcription factors) and *regulators* (i.e., transcription factors, TFs). Furtherly, the algorithm VIPER (Algorithm for Virtual Inference of Protein-activity by Enriched Regulon analysis) was used to inference protein activity and to identify the most significant TF regulators, which control the expression profile of the studied cells. Applying these algorithms, a footprint of candidate master regulators of BM-MSCs was defined, including the genes EPAS1, NFE2L1, SNAI2, STAB2, TEAD1, and TULP3, that presented consistent upregulation and hypomethylation in BM-MSCs. These TFs regulate the activation of the genes in the bone marrow MSC lineage and are involved in development, morphogenesis, cell differentiation, regulation of cell adhesion, and cell structure.

## 1. Introduction

Mesenchymal Stromal Cells (MSCs) are multipotent cells located in the stroma of multiple human tissues. In particular, they are present in the bone marrow (BM) hematopoietic niche, coexisting and regulating the maintenance of hematopoietic stem cells (HSCs). This BM niche also includes osteolineage cells (i.e., osteoblasts and osteoclasts), perivascular cells, endothelial cells, adipocytes, and macrophages. In this complex scenario, MSCs coordinate several critical activities, including self-renewal, mobilization, engraftment, and lineage differentiation [1]. 

When cultured in vitro, MSCs are characterized by their adherence to plastic; by their ability to differentiate in vitro to osteoblasts, adipocytes, and chondroblasts under specific culture conditions; and by the expression of a characteristic immunophenotypic profile, being positive for CD73, CD90, and CD105 marker genes and negative for CD34, CD45, CD14 or CD11b, CD19 or CD79α, and HLA-DR [2]. Close to MSCs, fibroblasts (FIBs) are non-stem cells that present a fairly similar phenotype to that of MSCs but differ in that they express other marker genes: CD10, CD26, CD106, and collagen VII (COL7A1). In addition, FIBs also share immunomodulatory proprieties with MSCs, such as the modulation of macrophages and the suppression of T cell proliferation [3]. Despite these similarities in phenotype, their transcriptomic signatures show significant differences between them, associating FIBs with a clear enrichment in genes related to the organization and function of the extracellular matrix and BM-MSCs in bone development tasks [4].

Due to their immunomodulatory ability, MSCs present great importance in the field of cellular therapy. Recent studies show the importance of the stimulation of MSCs to enhance their role in tissue repair and regeneration [5,6]. In addition, as it has been already mentioned, MSCs are being involved in the regulation of several relevant functions of the bone marrow. Nevertheless, it is still unclear how they perform such actions and what genes drive their activity at a regulatory level. 

Given the relevance of MSCs in the BM niche, the present work pursues the identification of genes which regulate the most important functions of MSCs and compromise the MSC lineage, acting as master regulators of this cell type. The master regulators (MR) are defined as transcription factors (TF) that differentially regulate groups of target genes (called *regulons*). MRs regulate specific gene sets, activating or repressing their expression and, in consequence, activating or inhibiting the function of the corresponding proteins. To disclose the MRs of MSCs, we first selected and analysed a compendium of samples corresponding to primary human MSCs isolated from healthy donors and expanded in culture and the multiple cell types related to them. Over this compendium, after proper normalization and integration of the datasets, we applied ARACNe (*Algorithm* *for the Reconstruction of Accurate Cellular Networks*) and VIPER (*Algorithm* *for Virtual Inference of Protein-activity by Enriched Regulon analysis*) to infer dependency from similar expression patterns between target genes and TFs based on mutual information [7,8], to generate gene bipartite regulatory networks, and to identify master regulators. We also performed differential expression analysis and expression profiling to complement the data obtained with ARACNe and VIPER. DNA methylation profiles of MSCs were also analysed to validate the signal derived from the transcriptomic profiles. All these integrative analyses allow us to establish potential *master regulators* of MSCs in the hematopoietic niche. These candidate TF regulators and their associated gene sets, as *regulons*, define regulatory units that drive the mesenchymal lineage, providing the cellular characteristics of the MSCs and determining the specific functions that they play in the bone marrow. 

## 2. Materials and Methods 

### 2.1. Multiple Cell Sample Series with Transcriptomic Data Collected and Unified in a Compendium Set

For the transcriptomic analysis of this work, 18 datasets were used to create a large uniform compendium of 264 human samples with genome-wide expression data. All datasets were downloaded from public database GEO (Gene Expression Omnibus, www.ncbi.nlm.nih.gov/geo/). The IDs of the 18 datasets integrated are GSE2666, GSE3823, GSE6029, GSE6460, GSE7637, GSE7888, GSE9451, GSE9520, GSE9593, GSE9764, GSE9894, GSE10311, GSE10315, GSE10438, GSE11418, GSE12264, GSE18043, and GSE46053. The specific samples selected from each one of these datasets are described in Supplementary Table S1, which also indicates the authors and year of each set. In all cases, the transcriptomic profiles were obtained merging data of *Affymetrix* platforms HG-U133 A and B and from platform HG-U133 Plus 2.0, all corresponding to Human Genome high density oligo microarrays. The expression signals from the probes of these microarrays were mapped to genes (Ensemble genes (ENSG) done as described in Reference [9]), using as costume CDFs the R annotation packages from *BrainArray* version 23 (http://brainarray.mbni.med.umich.edu). As indicated in Supplementary Table S1, the biological samples were originally obtained from 10 different cell types: 47 samples of hematopoietic stem cells (HSC), 10 of them isolated from bone marrow of healthy donors (BM-HSC); 9 samples of lymphocytes (LYM) as hematopoietic differentiated cells; 116 samples of mesenchymal stromal/stem cells (MSC) isolated from different tissues (50 isolated from bone marrow of healthy donors, BM-MSC); 27 MSCs stimulated with cytokines (stMSC), 6 of them stimulated with TGFβ and selected for the comparison with MSCs; 11 samples of skin-derived primary fibroblasts (FIB); 13 primary osteoblasts (OSTB); 23 stimulated osteoblasts (stOST); 12 osteoblasts derived by differentiation from MSCs (dOSTB); 3 adipoblasts derived by differentiation from MSCs (dADIP); and 3 chondroblasts derived by differentiation from MSCs (dCHON). The transcriptomic signal from all these samples was normalized, and the batch effect was corrected as described in detail in an earlier publication of our laboratory [4]. In Supplementary Table S2, we also provide the given acronyms and the names of the cells included in the compendium, indicating the number of samples of each cell type, those which are primary cells and those which are derived from bone marrow. In particular, with respect to the 50 samples of BM-MSCs selected for our study, we checked that, in each corresponding GEO dataset, the samples were isolated using the standard protocol called “Minimal criteria for defining multipotent mesenchymal stromal cells” (from The international Society for Therapy position statement) (as indicated in Reference [10]). This means in practical terms that all samples selected correspond to primary MSCs from bone marrow of healthy donors isolated in culture (in pass 2–5) and characterized by the presence of specific CD surface markers: at least positive for CD73, CD90, and CD105 and negative for CD34 and CD45.

### 2.2. Regulatory Networks Based on Mutual Information

Target-TF regulatory networks were generated from the transcriptomic expression matrix obtained for the 264 samples and for the about 16,000 human genes measured. This expression matrix was analysed using the algorithm ARACNe (*Algorithm for the Reconstruction of Accurate Cellular Networks*), which applies information theory (Mutual Information (MI)) to calculate dependency between TFs and gene targets, avoiding many indirect interactions that are generally found through co-expression methods that are less accurate [7]. ARACNe was implemented using the R packages: *minet* and *parmigene* [11,12]. The MI values were filtered to select only the ones corresponding to the regulatory events that occur between Transcription Factors (TFs, considered *regulators*), and the linked genes (i.e., the targeted genes, considered as *regulons*). To map the TFs in the gene matrix, we used a comprehensive list that included 1544 *Homo Sapiens* transcription factors obtained from the database AnimalTFDB version 2.0. [13].

### 2.3. Differential Expression Between Six Types of Human Cells Related to Bone-Marrow MSCs

The normalized gene expression matrix was also analysed to obtain the differential expression (DE) between the MSCs and other 5 related cell types. Four of them were primary cells isolated from healthy individuals: HSC, LYM, FIB, and OSTB. The others were MSCs stimulated with TGFβ (stMSC). Therefore, we created a subset of 93 samples, corresponding with 50 samples of BM-MSCs, 10 samples of HSCs, 9 samples of LYMs, 11 samples of FIB, 13 samples of OSTBs, and 6 samples of stMSCs. DE analyses were done using *limma* R package [14]. The comparisons were binary generating 6 groups: MSC-HSC, MSC-LYM, MSC-FIB, MSC-OSTB, MSC-stMSC, and stMSC-HSC. The selection of significant differentially expressed genes was done using a 5 % false discovery rate (FDR, that corresponded to adjusted *p*-value  ≤  0.05), plus the top 30 genes with most significant fold change (FC) in log2 for each group (up- and downregulated). 

### 2.4. Detection of Master Regulators in the Regulatory Networks 

Master Regulators were detected using the algorithm called VIPER (*Virtual Inference of Protein-activity by Enriched Regulon analysis*), implemented in R and Bioconductor [8]. This computational algorithm allows an accurate assessment of protein activity from gene expression data. The method uses the expression matrix and the regulatory network provided by ARACNe to perform an enrichment statistical analysis on every *regulon* [8] and to identify the most significant TFs associated with the regulatory models derived from the comparison of specific sample sets. Taking ***y*** as the value of MI returned by ARACNe, significant association TF-targets were filtered using as threshold = ***mean(y)***. Furthermore, using VIPER, we compared the same groups of samples as we did with *limma*, that were MSC-HSC, stMSC-HSC and MSC-LYM (as cell types related to the hematopoietic niche); MSC-FIB (as cell types related to the stroma); and MSC-stMSC and MSC-OSTB (as cell types related to the mesenchymal lineage that can be originated from MSCs). Moreover, VIPER algorithm included bootstrapping, testing 100 times subsets of the samples to find and identify the most stable regulators. The algorithm also calculates the pleiotropy of the TFs [8]. From these analyses, we selected the most significant TF regulators found with a *p*-value < 0.05. The comparisons that provided the best signal corresponded to cell types related to the hematopoietic niche (MSC-HSC, stMSC-HSC, and MSC-LYM). In contrast, the comparison with FIB, OSTB, and stMSC did not find many significant regulators (only 4 upregulated TFs were found with a *p*-value < 0.05). The top 10 most overexpressed TFs and the top 10 most repressed TFs found significant, considering the 6 pairwise comparisons, were selected. The regulatory networks generated with the selected TFs were visualized using *Cytoscape* [15]. A complementary analysis based in the enrichment of TFs in the selected list of genes was done using *iRegulon* tool [16]. We applied this tool to the list of all the genes included the networks produced with VIPER. The tool *iRegulon* allows the identification of enrichment in specific *transcription factors binding sites* (TFBS) within the list of genes explored by comparative analysis of the sequences of the promoters of a query list of genes against curated datasets [16]. The tool also finds the corresponding TFs associated to the TFBSs.

### 2.5. Gene Set Functional Enrichment Analysis

The regulatory gene sets generated (that include each TF regulator and the corresponding regulated genes as regulons) were selected to perform functional enrichment analyses. The methods applied to do the enrichments were (i) DAVID bioinformatics tool (https://david.ncifcrf.gov/), that includes a functional annotation enrichment and clustering analysis [17], and (ii) GeneTerm-Linker bioinformatics tool (http://gtlinker.cnb.csic.es/), that allows concurrent annotation and enrichment in several biological spaces in a unified way: GO Biological Process, GO Molecular Function, GO Cellular Component, KEGG Pathways, and InterPro Motifs and Domains [18].

### 2.6. DNA Methylation Analysis

To complement the gene regulatory analyses based on expression and transcriptomic profiling, we performed a global analysis of DNA methylation profiles of the CpG islands of the genes found as TF regulators in MSCs versus HSCs. This was done selecting and analysing three independent DNA methylation datasets of human bone marrow MSCs from healthy donors—GSE79695 (with 12 samples), GSE129266 (with 7 samples), and GSE87797 (with 6 samples)—compared with one DNA methylation dataset of human HSCs from healthy donors—GSE63409 (with 5 samples). The DNA methylation of all these samples was measured using Illumina Infinium HumanMethylation450 BeadChips (that corresponds to platform GPL13534 in the GEO database). This technology allows quantification of the global DNA methylation of the CpG islands across the genome based on the measurement of about 450,000 methylation sites per sample at single-nucleotide resolution. The raw data derived from these datasets was downloaded from GEO (https://www.ncbi.nlm.nih.gov/geo/) and analysed using algorithm *minfi* in R [19,20]. All these samples were preprocessed and integrated in a unified collection, after batch effect correction and normalization using the *combineArray* function from *minfi* [20], followed by the *preprocessIllumina* function also from *minfi* [19]. The technology of the Illumina HumanMethylation450 BeadChip consists of a two-color array that interrogates the methylation status of 485,512 methylation loci (mostly CpG sites but also a small number of cytosines outside of the CpG context), using bisulfite-converted DNA. For each methylation locus, two signals of interest are recorded: One signal measuring the amount of methylated DNA (Meth) and the other signal measuring the amount of unmethylated DNA (Unmeth). In principle, the proportion Meth/(Unmeth + Meth) is the methylation ratio (referred to as *beta* value) in the population of cells from which the DNA was extracted [19]. Being Meth the methylated gene loci and Unmeth the unmethylated gene loci, the *beta* value is established within a range of 0 to 1 (where 0 corresponds to complete no-methylated status and 1 corresponds to complete methylated status). After the global normalization of the samples described and the calculation of the *Beta* values, we looked for the methylation signal of the CpG islands of the top 20 gene TF regulators that were found overexpressed or repressed in the BM-MSCs, and we compared such signals with the methylation profiles of HSCs.

## 3. Results and Discussion

### 3.1. Differential Gene Expression Profiling of MSCs versus Related Cell Types

**Figure 1:**
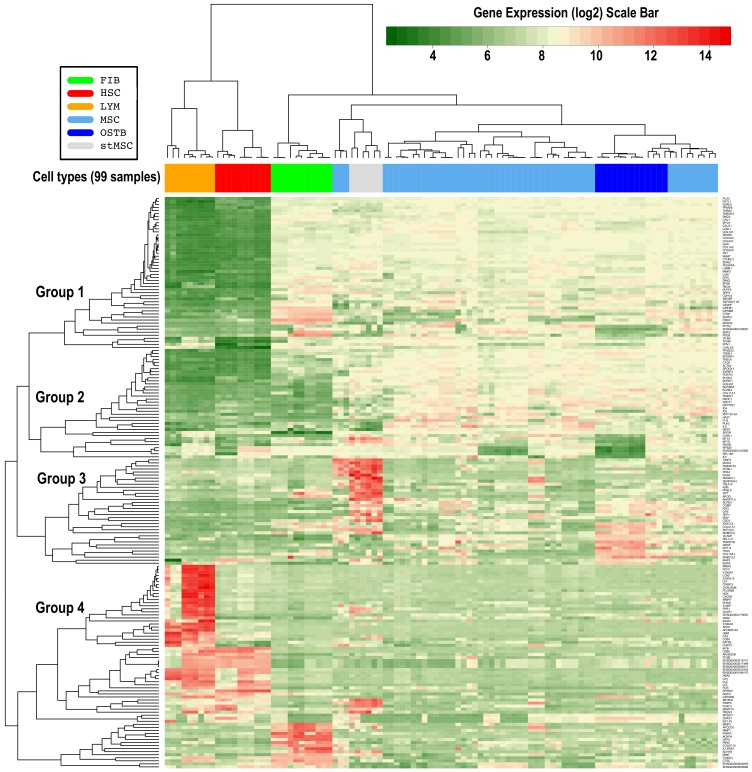
Heatmap presenting the expression profile of 188 genes found differentially expressed in the pair-wise comparisons of the six main cell types studied: Mesenchymal Stromal Cells (MSC), hematopoietic stem cells (HSC), fibroblasts (FIB), lymphocytes (LYM), osteoblasts (OSTB), and MSCs stimulated with cytokines (stMSC). The top 30 genes with most significant changes from each comparison were taken, and the union of these gave the list of 188 genes.

To gain insight into the transcriptomic characterization of MSCs, genome-wide expression profiles were generated for these mesenchymal cells isolated from bone marrow and several related human primary cells: hematopoietic stem cells (HSCs) and lymphocytes (LYMs) as cells of the hematopoietic lineage; fibroblasts (FIBs) as cells of the stromal lineage; primary osteoblasts isolated from bone (OSTBs); several cell types derived by differentiation from MSCs (adipoblasts dADIPs, chondroblasts dCHONs, and osteoblasts dOSTBs); and finally MSCs stimulated with TGFβ (stMSCs). After generating the global gene expression for the whole collection of 264 samples, as indicated in the Materials and Methods section, differential expression analyses were done for a subset of 99 samples to find the significative genes between 6 cell types of interest: MSC, HSC, LYM, FIB, OSTB, and stMSC. With these cells, the contrasts done were MSC-HSC, MSC-LYM, MSC-FIB, MSC-OSTB, MSC-stMSC, and stMSC-HSC. A differential expression signature of 188 genes corresponding to the top 30 most significant genes found in these six comparisons was produced, and these genes were used in the clustering expression analysis presented in Figure 1. The list of the 188 genes selected is also provided in Supplementary Table S3, that includes the statistical parameters corresponding to the differential expression analysis of MSCs versus HSCs. We also performed functional enrichment analyses of the selected set of 188 genes using GeneTerm-Linker and DAVID bioinformatic tools (as described in the Materials and Methods section). Gene expression clustering and gene functional association allow grouping the genes finding relevant links.

Upon examining specific genes in more detail, we found COL1A2, COL3A1, COL5A2, COL6A3, FN1, and CTGF among top 30 differentially expressed genes between MSCs and hematopoietic lineage (which includes HSCs and LYMs). These genes are common in extracellular matrix functions as well as in focal adhesion and skeletal system development and collagen binging. In fact, several of these genes take part in the PI3K-AKT pathway and interact with receptors and with the extracellular matrix. These interactions lead to direct or indirect control of cellular activities such as adhesion, migration, and differentiation. The differential signature of MSCs versus hematopoietic also contains numerous epithelial–mesenchymal transition (EMT) markers, such as the EMT-inducing gene SNAI2, and others: ACTA2, MMP2, and POSTN [21]. The genes COL5A2 and FN1 were also upregulated in stMSC and OSTB, indicating that upregulation of these genes is characteristic of the mesenchymal lineage. In the comparison between MSCs and stMSCs, it is important to consider that the stimulation of the cells (stMSCs) was done using TGFβ (the transforming growth factor beta-3), that is a cytokine involved in cell differentiation, embryogenesis, and development. Moreover, this gene is involved in cellular adhesion and ECM formation during the process of human embryonic palate development and regulates the movements of epidermal and dermal cells in injured skin [21]. In this regard, A2M, APOD, COMP, CPE, DPT, PRELP, SERPINA3, SPARCL1, and SPP1 are genes found upregulated in stMSCs in the contrast with MSCs, and all of them are annotated to the functions of differentiation and epithelial–mesenchymal transition (EMT), showing a high expression due to the addition of TGFβ [22,23]. Other genes such as FLG, IL6, MEST, PLK2, and VCAM1 were found upregulated in MSCs in contrast with stMSCs. These genes are involved in cell-cycle control. In particular, IL6 and PLK2 regulate JAK/STAT and p53 signalling pathways, respectively, to maintain the homeostasis and quiescent cellular status [24,25]; this could be the reason for the upregulation of the MSC in contrast to stimulated or differentiated MSCs. The cluster linked to OSTBs, includes genes with functions related to immunoregulation, such as CSF2RB, IGDCC4, NDNF, and VCAM1, and genes related with ECM, such as COL15A1, COL21A1, and ITIH5. Moreover, other genes in this cluster are EGFR2 and GREM1, which are genes necessary for the skeletal development and bone homeostasis and are also activated during bone cells response to mechanical strain [26,27]. 

All the genes mentioned in this section are included in the heatmap presented in Figure 1. To facilitate the location of the genes, the dendrogram has been divided in 4 groups and the genes in each group can be found in Supplementary Figure S4, which includes an enlarged version of the dendrogram with readable gene names.

In the comparison of MSCs with FIBs, even though significant differences were found for genes like CHI3L1, FNDC1, POSTN, SORBS2, SRGN, TM4SF1, and VCAM1; these genes are usually identified as expressed in fibroblasts, since they contribute to the structural integrity of the extracellular matrix, showing high similar functions in the stromal lineage (where FIBs and MSCs are included). Other genes, such as COL1A2, COL3A1, COL6A3, FN1, GJA1, and LUM were also present in both cell types, presenting a high expression in comparison with hematopoietic cell lineages. These genes are functionally related to focal adhesion, involving the PI3K-AKT signalling pathway and the extracellular matrix (ECM)–receptor interaction. 

The heatmap (Figure 1) also includes other genes, such as ARHGBID, CD69, FOSB, HLA-DRB1 (ENSG00000206241), MYB, and SPINK2, which are a group of the most differentiated genes overexpressed in HSCs, forming an expression cluster associated to the HSCs. Moreover, many of them are consistently associated with gain and activation of hematopoietic-specific enhancers [28]. CD69 and HLA-DRB1 are genes that play a central role in the immune system and are constitutively expressed in T cells. This result is important since CD69 has been described as heterogeneous in HSCs, and our observation marks this gene as important for the mobilization and activation of stem cells in hematopoietic differentiation [29,30]. 

### 3.2. Identification of Master Regulators of MSCs and of Other Related Cell Types

**Figure 2:**
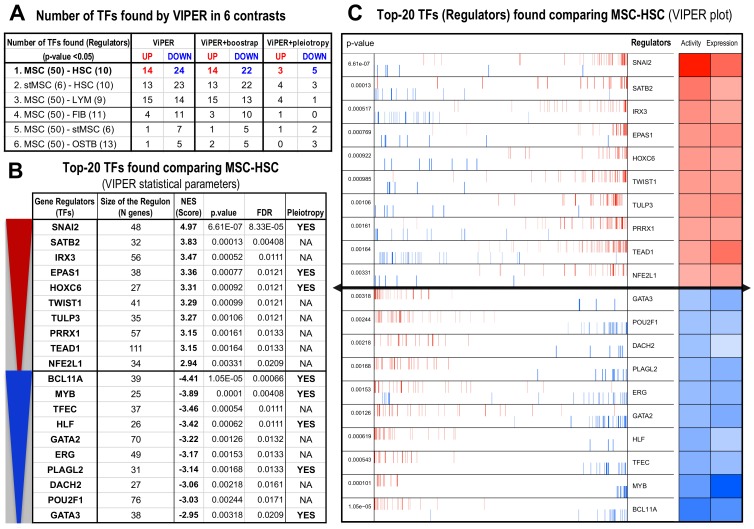
Gene *regulators* (TFs) and associated gene *regulons* found using VIPER (Algorithm for Virtual Inference of Protein-activity by Enriched Regulon analysis) in the comparison of the expression profiles of MSCs versus the other 5 related cell types, most of them present in the human bone marrow: (**A**) Table showing the number of TFs up- or downregulated and found in each comparison; (**B**) table with the top 10 up- and downregulated TFs found, including the parameters provided by VIPER (normalized enrichment score (NES), *p*-value, false discovery rate (FDR), and pleiotropy); and (**C**) plot produced by VIPER presenting the top 20 upregulated (red) and downregulated (blue) TFs that illustrates the strength of the protein activity and the RNA expression (darker colors higher values).

To identify the possible role of the master regulators in MSCs, we used the ARACNe and VIPER algorithms [7,8]. The comparative analysis, based in VIPER, was done for the same sample groups used in the *limma* differential expression, that were MSC-HSC, stMSC-HSC, MSC-LYM, MSC-FIB, MSC-stMSC, and MSC-OSTB. The number of master regulators found in these 6 comparisons (five contrasts against MSCs and another contrast between stMSC and HSC) is presented in Figure 2A. 

The analyses indicate that some comparisons detect many more changed TFs than others, revealing the dissimilarity or distance between the cell types. In this way, the results show major changes: (i) for MSC versus HSC (with 14 upregulators and 22 downregulators found after the bootstrapping analysis), (ii) for stMSC versus HSC (13 upregulators and 22 downreglulators), and (iii) for MSC versus LYM (15 upregulators and 13 downregulators). By contrast, due to the similarities between MSC, FIB, OSTB, and stMSC, in the case of MSC versus FIB, only 3 upregulators and 10 downregulators appear, and this decreases more in the comparisons MSC-OSTB and MSC-stMSC, with 2 and 1 upregulators plus 5 downregulators, respectively (Figure 2A). The table also shows the number of TFs found considering pleiotropic effects due to the confluence of several genetic traits. This analysis greatly reduces the number of regulators since it implies cooperativity.

As a whole, the results identified the most relevant functional differences between the lineages mesenchymal and hematopoietic. Consequently, the genes found in the contrasts of MSCs versus HSCs were analysed in more detail. Figure 2B shows the top 10 gene upregulators—SNAI2, STAB2, IRX3, EPAS1, HOXC6, TWIST1, TULP3, PRRX1, TEAD1, and NFE2L1—and top 10 gene downregulators—BCL11A, MYB, TFEC, HLF, GATA2, ERG, PLAGL2, DACH2, POU2F1, and GATA3—common in all the three contrasts between these lineages (1, 2, and 3 in Figure 2A). Interestingly, the size of the regulon (*N* genes) indicates the number of target genes that were found associated with each master regulator. In the master upregulators, TEAD1 presents the largest number with 111 genes, followed by PRRX1 (57) and IRX3 (56). In the case of the master downregulators, POU2F1 (76) and GATA2 (70) have the largest number of associated genes. The table in Figure 2B also presents in which genes there is pleiotropy: SNAI2, EPAS1, and HOXC6 in the case of upregulators and BCL11A, MYB, HLF, PLAGL2. and GATA3 in the case of downregulators. With respect to the biological functions, the top 10 TFs upregulated in MSCs were involved mainly in morphogenesis and development functions and the top 10 TFs upregulated in HSCs presented mainly functions related to hematopoiesis, lymphocytes formation, and immune system regulation (see Table 1).

Figure 2C corresponds to the enrichment plot with the top 20 TF master regulators found in our analysis. The colour panels on the right represent (i) *expression*, the expression value of each TF in the dataset, and (ii) *activity*, the estimated protein activity corresponding to this regulator in the system. These parameters provide a measure of the importance of these genes to shape the biological characteristics of the MSCs and the mesenchymal lineage. SNAI2 is the most significant upregulator gene and has been described as a master TF in organogenesis and wound healing and is directly involved in the epithelial to mesenchymal transition (EMT) [31]. The highest activity in downregulators of MSCs was observed for BCL11A and MYB. The MYB gene is an important regulator of hematopoietic cell development and plays a central role in cell-cycle progression in B- and T-lymphoid progenitor cells [32].

### 3.3. Differential Expression Analysis of the MSCs Master Regulators

To support the characterization of the top 20 master regulators found with VIPER in the comparison of BM-MSCs with other cell types, we elaborated a parallel differential expression analysis (using *limma*) with the cells described before (performing the same 6 comparisons of cells, Figure 2A). In the results of these DE analyses, we searched for the changes of the 20 TFs found with VIPER. The numbers corresponding to the log2 of the fold change (log2FC) observed for each one of these 20 TFs are presented in Supplementary Table S5. The fold change represents a good measure of the intensity of the changes. Corroborating the results, the gene SNAI2 is found the most upregulated with a high differential expression in MSCs with respect to the hematopoietic lineage (compared to HSCs log2FC = 5.93 and compared to LYMs log2FC = 6.49), while MYB is found the most in the opposite case (log2FC = –6.04 compared to HSCs and log2FC = –3.62 compared to LYMs). In the case of the comparisons between cell types that belong to the mesenchymal or stromal lineage (which are MSCs, stMSCs, FIBs, and OSTBs), the top 20 TFs do not show large changes and the values of log2FC are always between 1 and –1 (i.e., close to 0). Such small changes reflect that these cell types are closer in biological genetic terms. For example, PRRX1 is a paired-related homeobox (PRRX) transcription factor that regulates mesenchymal cell fate and stands at the centre of a network coordinating fibroblast differentiation; therefore, it is described as a regulator of FIBs that also regulates MSCs during the development [33]. By contrast, IRX3 is not so similar for all the mesenchymal lineage cells, showing a clearer overexpression in MSCs with respect to FIBs (log2FC = 2.13).

Related to the TFs downregulated in MSCs, it may be interesting to remark the case of HLF, which acts as a negative activator, because HLF is a relevant TF in some leukemia due to TCF3-HLF fusion protein, which suppresses RUNX1 transcription and activates expression of LMO2 and several *Groucho-related* genes as well as antiapoptotic genes like SNAI2 [34].

### 3.4. Methylation Profiles of the MSCs Master Regulators

**Figure 3:**
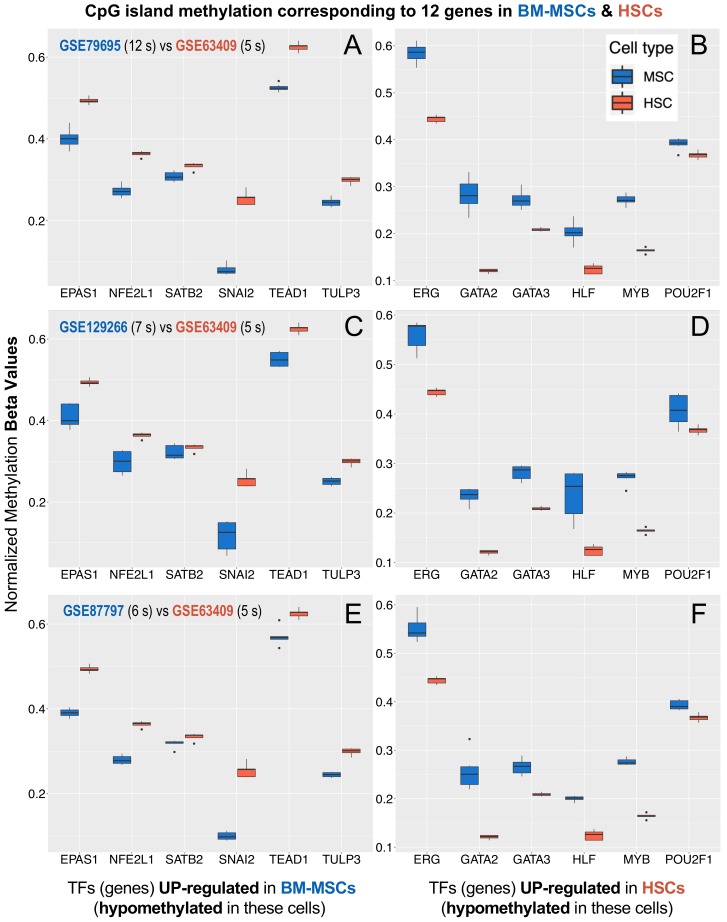
DNA methylation levels (measured as Beta values) of 12 TFs (blue boxplots) in 3 independent datasets of BM-MSCs (**A**,**C**,**E**) (GSE79695, GSE129266, GSE87797) in comparison with the methylation levels of the same TFs (red boxplots) in a dataset of HSCs (**B**,**D**,**F**) (GSE63409) (s indicates the number of samples). The TFs represented are: EPAS1, NFE2L1, SATB2, SNAI2, TEAD1, TULP3 hypomethylated in BM-MSCs; and ERG, GATA2, GATA3, HLF, MYB, POU2F1 hypermethylated.

As an independent validation of the top 20 MSC master regulators found in the gene regulatory analysis performed with VIPER and corroborated by the differential expression analysis performed with *limma*, we analysed the methylation profiles of the promoters of these TF genes to verify whether they correlated with their relative expression signal. To do this, as outlined in the Materials and Methods section, we used three independent DNA methylation profiles of BM-MSCs and compared them with a DNA methylation profile obtained for HSCs. After adequate robust normalization of the datasets (as described in the Materials and Methods section), differential methylation of the CpG islands of these genes was determined and compared as presented in Figure 3. The results provided relative hypomethylation for the genes EPAS1, NFE2L1, SATB2, SNAI2, TEAD1, and TULP3 from our list of upregulated TFs and relative hypermethylation for ERG, GATA2, GATA3, HLF, MYB and POU2F1 from our list of TF downregulated in BM-MSCs (Figure 3).

The methylation results provide special support to the SNAI2 gene as a master regulator of BM-MSCs. A study with fibroblasts showed that the associated proximal promoters of SNAI1 and SNAI2 genes were hypomethylated due to EMT, being characteristic of stromal cells [35]. Furthermore, the association between DNA methylation and transcription levels for the SNAI2 gene has been demonstrated in iPSC (induced pluripotent stem cells) generated from fibroblasts [35]. Hypomethylation of TEAD1 gene, also found in our analysis, has been reported to be a requirement for the ability of MSCs to undergo proper differentiation [36]. These studies also suggested that aberrant DNA hypermethylation of the loci of genes of the TEAD family could compromise their role in the development of BM-MSCs and could promote malignant diseases originated in the bone marrow, such as multiple myeloma (MM) [36]. EPAS1 gene was also detected in our results hypomethylated in BM-MSCs, and this gene showed a decrease in repressed chromatin marks in mesenchymal stromal cells isolated from bone marrow and adipose tissue, suggesting that epigenetic mechanisms are probably involved in determining the stem potential of MSCs via this gene [37]. In addition, our data also showed a decreasing in methylation of the gene locus of SATB2 (special AT-rich sequence-binding protein 2). In this sense, regulatory studies have shown that the activity of SATB2 is modulated epigenetically and that, when this gene shows decrease in methylation, this triggers bone marrow stromal cells osteogenic differentiation, facilitating bone formation and regeneration [38]. All the reports referenced in this section and our results in the search for master regulators confirm that epigenetic regulation of gene expression is a central mechanism that governs cell stemness, determination, commitment, and differentiation. Taken together, these results reinforce our findings of candidate master regulators of BM-MSCs.

### 3.5. Construction of Bipartite Networks Including Regulators and Regulons

**Figure 4:**
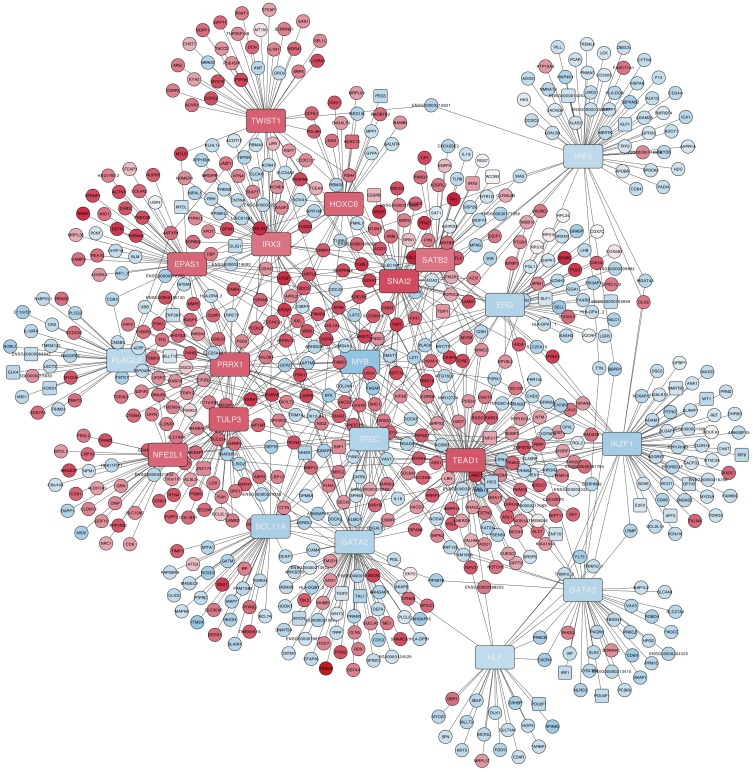
Gene co-regulation network presenting the top 10 up- and top 10 downregulated master regulators (in red and blue rectangles, respectively): The master regulators are connected with their regulons. Upregulated genes have a red background, while downregulated genes have a pale blue color.

**Figure 5:**
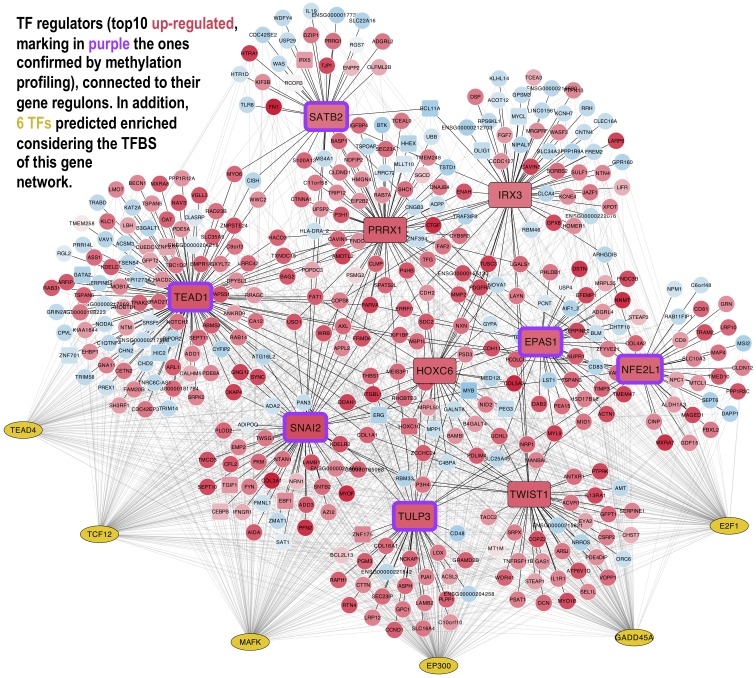
Gene co-regulation network presenting the top 10 upregulated master regulators (red rectangles): The master regulators are connected with their regulons that are the gene sets that each one regulates: Upregulated genes have a red background, while downregulated genes have a pale blue color. The 6 TFs (E2F1, EP300, GADD45A, MAFK, TCF12, and TEAD4) included in yellow ellipses correspond to the TFs found enriched with the *iRegulon* tool in the promoters of all the genes of the network.

Using the links found with ARACNe between each gene regulator and its target genes, we can build regulatory networks including all the most stable and significant regulators and their genes, marking also the expression level detected. These regulatory networks are represented as a bipartite graph that includes two types of nodes (regulator TFs and regulon targets) plus directed links between them (TF to Target). Figure 4 and Figure 5 present two views of these type of networks: first (Figure 4) including the top 20 regulators (10 up- and 10 downregulated) linked to their gene regulons (presenting the nodes in red when they are upregulated and in blue when they were downregulated); second (Figure 5) including only the top 10 upregulated TFs linked to their regulons (again presenting the upregulated nodes in red and the downregulated nodes in blue). 

In both networks, the intensity of the colour of the nodes is proportional to the expression signal values of the corresponding genes. In the case of the second network (Figure 5), 6 other TFs were found using the *iRegulon* tool over the entire list of genes and TFs of VIPER (as indicated in the Materials and Methods section) [16].

The network in Figure 4 shows the central regulatory role of SNAI2. This gene regulates important genes (such as CEBPB, EBF1, ERG, MYB, TGIF1, and ZMAT1), which are TFs in charge of DNA-binding transcriptional activation and transcription regulation. Other meta-regulators are TEAD1, which regulates TFs related to phosphorylation such as COPS6, KUF1BP, MYO6, and RGL2; and TULP3, which shows the regulation of TFs as CCND1, LAMB2, and THBS1, and regulates the PI3K/AKT signalling pathway that performs a critical role in regulating diverse cellular functions including metabolism, growth, proliferation, survival, transcription, and protein synthesis as well as in extracellular matrix organization, ECM–receptor interactions, cell adhesion, and integrin binding. 

With respect to the TFs found repressed, MYB shows the strongest signal both in the regulatory network derived from VIPER and in the differential expression analysis, and it was found hypomethylated in HSCs. MYB regulates TFs related to cellular transport (such as AP2M1, ATP8A1, KDELR1, LAPTM5, TMED10, and UCP2) and regulates other TFs responsible of the cell proliferation (such as EPS8, SYK, and YAP1). The role of TFs that are downregulated in MSCs versus HSCs may reveal a positive action or a more relevant function in the hematopoietic lineage.

Figure 5 presents the regulatory network produced by selecting only the top TF regulators that were overexpressed in MSCs. These TFs are presented in large red rectangles in the figure. Those marked with a purple frame were the ones that also showed a low methylation level in the analysis of the CpG islands of these genes in BM-MSCs (as shown in Figure 3). Furthermore, other 6 TFs (E2F1, EP300, GADD45A, MAFK, TCF12, and TEAD4) are included in Figure 5 in yellow ellipses and correspond to the TFs found enriched in the promoters of all the genes of this network. A search of public data revealed that E2F1, EP300, GADD45A, and SPL1 are highly expressed in bone marrow and that E2F1, EP300, and GADD45A are genes expressed in response to the detection of DNA damage and can cause a the reduction of the cell cycle rate, characteristic of the quiescent state of stem cells.

### 3.6. Functional Enrichment Analysis of the Master Regulators and Their Regulons 

The results of the functional enrichment analyses are presented in Table 1. The analysis was done first with the list of upregulated TFs and their gene regulons (marked UP in Table 1) and second with the list of downregulated TFs and their gene regulons (marked DOWN in Table 1). The upregulated TFs and their regulons show first a significant enrichment in functions associated to the nervous system: generation of neurons, neurogenesis, and nervous system development, being IRX3, SATB2, TULP3, and TWIST1 the master regulators included in these functions (the complete list of all the genes associated to each enriched function is included in Supplementary Table S6). The classical differentiation paths of MSCs are adipogenesis, chondrogenesis, and osteogenesis, but recent studies show that MSCs have also the plasticity to differentiate into cells of ectodermic origin like neurocytes [39]. Moreover, TULP3 regulates hedgehog signalling pathway and promotes the development of multipotent neural crest progenitors endowed with both mesenchymal and neural potentials [40]. Functional enrichment on organ morphogenesis and development is the second most significant biological trait found in the regulators and regulons overexpressed in MSCs. These biological features involve again as master regulators SATB2 and TULP3 and bring together EPAS1, TEAD1, and SNAI2. Endothelial PAS domain Protein 1 (EPAS1) promotes adipose differentiation and is a TF specific in endothelial cells as an important regulator of vascularization. These functions are related to the role and action of MSCs inside the bone marrow, indicating that the regulators found are essential to the function of MSCs [41]. All these TFs were found to be overexpressed and hypomethylated in MSCs, being postulated as key master regulators of this cell linage.

Regarding the top 10 master regulators that are downregulated in MSCs and upregulated in HSCs, many of them have hematopoietic functions: BCL11A and IKZF1 are strongly related to hematopoiesis and to the immune system. IKZF1 was described as a regulator of gene expression and chromatin remodelling, playing an important role in the correct development of the immune system and acting as an important tumour suppressor in lymphoblastic leukaemia (ALL) [42]. BCL11A is also important in hematopoiesis, with a particular role in B-cell development and in the maintenance of stemness in HSCs. Besides, BCL11A is highly expressed in the initial phases of myeloid and lymphoid malignancies, indicating that a high level of BCL11A can cause leukaemia cells’ continuous replication, blocking differentiation [43]. All these data suggest that the master regulators found repressed are responsible for the maintenance of the hematopoietic cell lineage. 

## 4. Conclusions

Transcription factors are known to maintain stemness and to drive differentiation of cell lineages. Our study presents a selection of relevant master regulators that define human primary MSCs, classifying different groups of related cell types and providing a transcriptomic footprint with the most relevant gene regulators and regulons. Likewise, we found links between the MSCs and gene-regulating extracellular matrix functions as well as other functions, such as adhesion, migration and differentiation, and maintenance of the BM niche. Many of these functions are also directly related to the epithelial–mesenchymal transition (EMT), a process by which epithelial cells lose their cell polarity and cell–cell adhesion and gain migratory and invasive properties to become mesenchymal stem cells or cells of the mesenchymal lineage. Our finding of SNAI2 as one of the master regulators of MSCs gives strong support to the functional link of these cells with the EMT. 

This article also highlights the importance of the specific links and relationships between master regulators, forming networks, to clarify the complexity and cooperation between them beyond the individual regulation of each one in MSCs. As an example of these interactions, SNAI2, SATB2, and TULP3 have been identified as a group of relevant upregulated master regulators, being involved in the maintenance of MSCs through Hedgehog and PI3K/AKT signalling pathways, that are essential to induce stem cell traits, immunosuppression, senescence, drug resistance, and metastasis. With respect to previous studies, TULP3 has not been directly related to MSCs. By contrast, SNAI2 has previously been associated with MSC, involved in ECM organization, and functionally associated with EMT. Among the downregulated master regulators, MYB and BCL11A have been related to immune system regulation in hematologic malignancies, suggesting their participation in the maintenance of the hematopoiesis and in the regulation of other downregulated master regulators such as IKZF1. Finally, a complementary epigenetic study was carried out to obtain the DNA methylation profiles of the TFs found, which corroborated the gene expression profiles and gave support to EPAS1, NFE2L1, SATB2, SNAI2, TEAD1, and TULP3 as candidate positive master regulators of MSCs.

To summarise, the work presents a set of transcription factors associated to the mesenchymal lineage as well as their direct links with other regulators and other genes, deciphering the regulatory networks of the human BM-MSCs. For future work, we are interested in developing new trials and tests with the top 10 overexpressed master regulators found in this work to investigate their modulation in different contexts and to better understand the dynamic behaviour of MSCs within the hematopoietic niche, with a particular focus on their immunodulatory properties. 

## Figures and Tables

**Table 1:** Functional enrichment analysis done with the top 10 master regulators (TFs) found upregulated (UP) and their corresponding gene regulons and with the top 10 master regulators (TFs) downregulated (DOWN) and their corresponding gene regulons. The genes asigned to each function are included in Supplementary Table S6.

Enriched Functional Term	N Genes (in the Function)	N Genes (in the Query)	N in Function/N in Query (%)	Regulation (UP/DOWN)	*p*-value (adj. Benjamini)
**generation of neurons**	**26**	283	7.34	**UP**	0.015168
**neurogenesis**	**30**	283	8.47	**UP**	0.001735
**nervous system development**	**42**	283	11.86	**UP**	0.007296
**cell-substrate adhesion**	**10**	283	2.82	**UP**	0.014732
**extracellular matrix**	**22**	323	6.21	**UP**	0.001305
**cytoskeleton**	**51**	323	14.41	**UP**	0.003200
**face morphogenesis**	**4**	283	1.13	**UP**	0.047319
**embryonic development**	**29**	283	8.19	**UP**	0.001836
**organ morphogenesis**	**31**	283	8.76	**UP**	0.000405
**organ development**	**57**	283	16.10	**UP**	0.016957
**cell differentiation**	**61**	283	17.23	**UP**	0.000488
**T cell activation**	**17**	293	5.00	**DOWN**	0.000004
**lymphocyte activation**	**21**	293	6.18	**DOWN**	0.000005
**hemopoiesis**	**22**	293	6.47	**DOWN**	0.000010
**hemopoietic or lymphoid organ development**	**22**	293	6.47	**DOWN**	0.000043
**immune system development**	**22**	293	6.47	**DOWN**	0.000074
**regulation of immune system process**	**23**	293	6.76	**DOWN**	0.002043
**calponin-like actin-binding**	**10**	323	2.94	**DOWN**	0.002577
**actin filament-based process**	**19**	293	5.59	**DOWN**	0.000509
**cytoskeleton organization**	**26**	293	7.65	**DOWN**	0.000664
**GTPase regulator activity**	**24**	304	7.06	**DOWN**	0.001932

## Supplementary Materials

The following files are available online at https://www.mdpi.com/2218-273X/10/4/557/s1. Supplementary Table S1: List of human cells used in this study that correspond to 18 datasets including a compendium of 264 human samples with genome-wide expression data: All datasets were downloaded from the public database GEO (Gene Expression Omnibus, www.ncbi.nlm.nih.gov/geo/). The IDs of the 18 datasets integrated are GSE2666, GSE3823, GSE6029, GSE6460, GSE7637, GSE7888, GSE9451, GSE9520, GSE9593, GSE9764, GSE9894, GSE10311, GSE10315, GSE10438, GSE11418, GSE12264, GSE18043, and GSE46053. The specific number of samples selected from each one of these datasets are indicated within the table. Supplementary Table S2: List of 10 human cell types used in this study, indicating the ones that were primary cells, stimulated cells, or cells derived from MSCs. The set of 93 samples is the one included in the comparisons done with VIPER algorithm for regulation and with *limma* algorithm for differential expression. Supplementary Table S3: List of 188 genes included in the heatmap presented in Figure 1 of the article: The table presents for these genes the results of the differential expression analysis of BM-MSCs (50 samples) versus HSCs (10 samples) done with *limma*: log2 fold-change; mean expression of the gene across all the samples; F-statistic; raw *p*-value; and adjusted *p*-value. This list of genes was composed selecting the top 30 genes that provided the most significant differences in each one of the 6 comparisons of cell types performed in this work: MSC-HSC, stMSC-HSC, MSC-LYM, MSC-FIB, MSC-stMSC, and MSC-OSTB. Supplementary Figure S4: Dendrogram divided into 4 groups corresponding to the genes included in the heatmap of Figure 1, presented in an enlarged version with readable gene names to facilitate the location of each gene. Supplementary Table S5: Results of the differential expression (DE) analysis done with *limma* corresponding to the top 20 gene regulators (TFs) found in this work: The data correspond to the DE parameters (F-statistic, raw *p*-values, and adjusted *p*-values) of the comparison of MSCs versus HSCs. The log2FoldChanges of all the 6 comparisons (MSC-HSC, stMSC-HSC, MSC-LYM, MSC-FIB, MSC-stMSC, and MSC-OSTB) are also included. The mean expression of each of the 20 genes in all samples is also presented. Supplementary Table S6: Functional enrichment analysis (i) done with the top 10 master regulators (TFs) found upregulated (UP) and their corresponding gene regulons and (ii) done with the top 10 master regulators (TFs) downregulated (DOWN) and their corresponding gene regulons. The genes marked in bold in the column “Genes” correspond to TFs.

## Abbreviations

BM, Bone Marrow; CDF, Chip Description File; CpG, CG sequence islands in the genome; GO, Gene Ontology; HSC, Hematopoietic Stem Cell; KEGG, Kyoto Encyclopedia of Genes and Genomes; MSC, Mesenchymal Stromal/Stem Cell; TF, Transcription Factor.
