# Supplementary figures and images for: Deciphering Master Gene Regulators and Associated Networks of Human Mesenchymal Stromal Cells
Source: Biomolecules. 2020 Apr 5;10(4):557. doi: 10.3390/biom10040557 (PMC7226324; doi:10.3390/biom10040557)

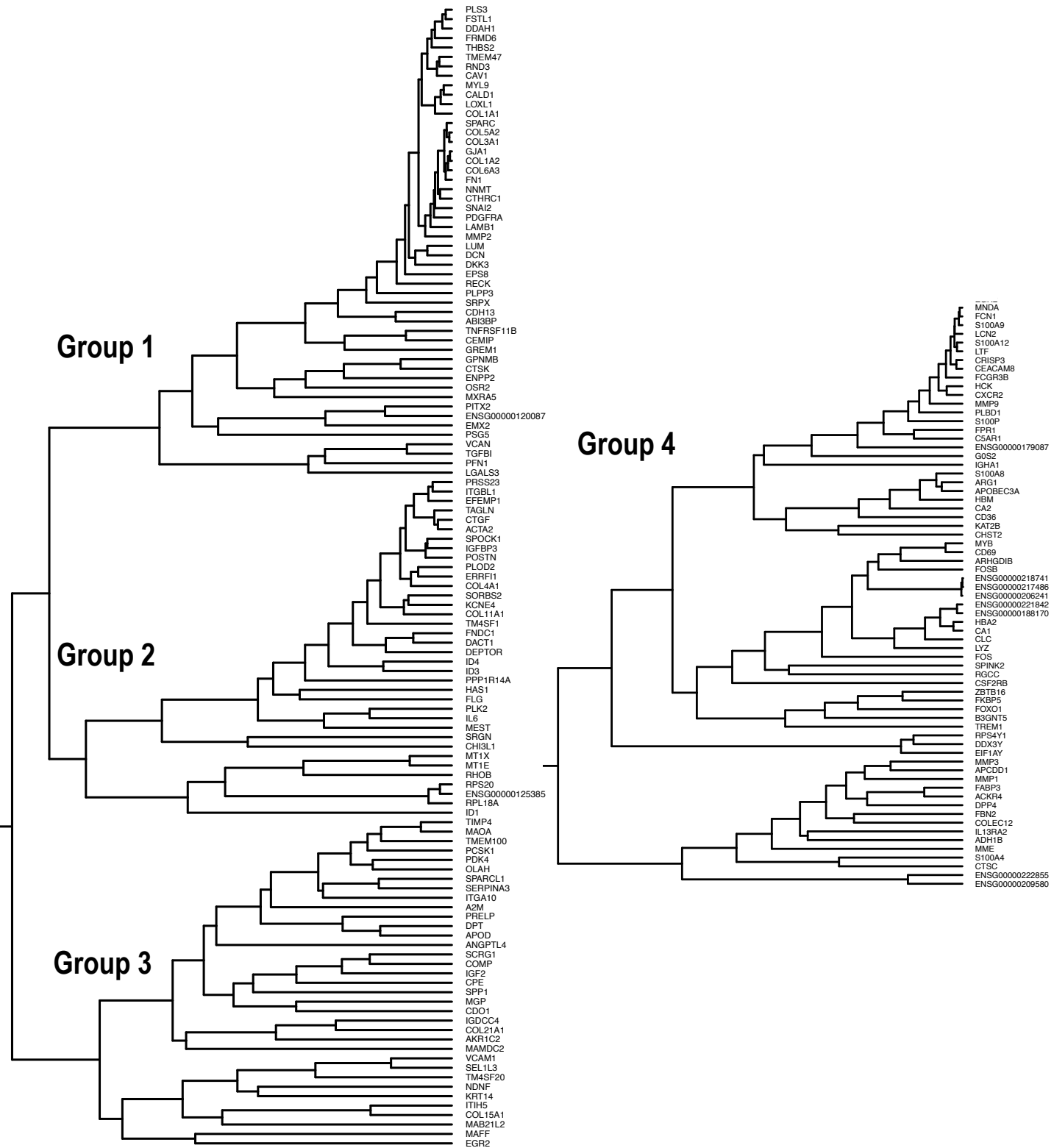

Supplement: Supplementary file 1 [file biomolecules-10-00557-s001.zip › SanchezLuisEetal_Reviewed1_Supp_FIGURE-S4.pdf]
